# Spatial differentiation of gene expression in *Aspergillus niger* colony grown for sugar beet pulp utilization

**DOI:** 10.1038/srep13592

**Published:** 2015-08-28

**Authors:** Isabelle Benoit, Miaomiao Zhou, Alexandra Vivas Duarte, Damien J. Downes, Richard B. Todd, Wendy Kloezen, Harm Post, Albert J. R. Heck, A. F. Maarten Altelaar, Ronald P. de Vries

**Affiliations:** 1 Fungal Physiology, CBS-KNAW Fungal Biodiversity Centre, Uppsalalaan 8, 3584 CT Utrecht, The Netherlands; 2 Microbiology & Kluyver Centre for Genomics of Industrial Fermentations, Utrecht University, Padualaan 8, 3584 CH, Utrecht, The Netherlands; 3 Fungal Molecular Physiology, Utrecht University, Utrecht, The Netherlands; 4 Department of Plant Pathology, Kansas State University, 4024 Throckmorton Plant Sciences Center, Manhattan, KS 66506, USA; 5 Biomolecular Mass Spectrometry and Proteomics, Bijvoet Centre for Biomolecular Research and Utrecht Institute for Pharmaceutical Sciences, Utrecht University, Padualaan 8, 3584 CH Utrecht, The Netherlands

## Abstract

Degradation of plant biomass to fermentable sugars is of critical importance for the use of plant materials for biofuels. Filamentous fungi are ubiquitous organisms and major plant biomass degraders. Single colonies of some fungal species can colonize massive areas as large as five soccer stadia. During growth, the mycelium encounters heterogeneous carbon sources. Here we assessed whether substrate heterogeneity is a major determinant of spatial gene expression in colonies of *Aspergillus niger*. We analyzed whole-genome gene expression in five concentric zones of 5-day-old colonies utilizing sugar beet pulp as a complex carbon source. Growth, protein production and secretion occurred throughout the colony. Genes involved in carbon catabolism were expressed uniformly from the centre to the periphery whereas genes encoding plant biomass degrading enzymes and nitrate utilization were expressed differentially across the colony. A combined adaptive response of carbon-catabolism and enzyme production to locally available monosaccharides was observed. Finally, our results demonstrate that *A. niger* employs different enzymatic tools to adapt its metabolism as it colonizes complex environments.

Filamentous fungi live in nearly all ecosystems on Earth and play a major role in decomposing organic material. The fungal colony consists of interconnected hyphae and can cover immense areas. The largest and oldest living organism belongs to the genus Armillaria^1^. *Armillaria ostoyae,* for instance, extends over approximately 37 ha^2^. Taking into account the complexity of the environment and the changes in available carbon sources during the course of fungal growth, the mycelium of a single colony encounters a heterogeneous substrate^3^. The periphery of the colony is exposed to unexplored organic material as the hyphae extend during growth, whereas the colony center directly contacts a substrate that has been partially utilized. Previous studies on *Aspergillus niger* grown on xylose or maltose have shown that the vegetative colonizing mycelium is highly differentiated for enzyme secretion^4^. Some exploring hyphae strongly secrete glucoamylase when others do not. Moreover, on xylose or maltose, mycelial growth and protein secretion mainly take place at the periphery of the colony^5^. When the same colony is transferred to fresh medium, protein secretion occurs at the periphery and throughout the colony. When growing in their natural biotope, fungal colonies are not exposed to carbon depletion, but instead different parts of the mycelium are exposed to heterogeneous carbon sources with variable composition. To study differentiation of gene expression in a more “natural” situation, we grew *A. niger* on a crude plant biomass substrate, sugar beet pulp (SBP).

*A. niger* is one of the most important industrial fungi worldwide and is a model organism for plant biomass utilization, enzyme secretion and carbon metabolism in fungi. Sugar beet (*Beta vulgaris*) is the main crop for sugar production in Europe (http://epp.eurostat.ec.europa.eu). In United States, sugar beet has provided about 55 percent of the total sugar produced domestically since the mid-1990 s (http://www.ers.usda.gov). SBP is the cell debris that remains after sugar extraction from the sugar-beet roots. SBP is a well-known source of pectin as 24–32% of the biomass is pectic substances, but it also contains 24–32% hemicellulose, 22 to 40% cellulose, 3–5% lignin and 8–11% proteins^6^,^7^. Therefore sugar beet pulp waste from the sugar industry is a potential source of carbon for biofuel production, provided that these complex carbon molecules can be efficiently degraded^8^. In this study we investigated how *A. niger* uses its enzymatic toolbox throughout the vegetative mycelial colony to degrade sugar beet pulp.

## Results

### Protein synthesis and secretion show distinct patterns across the *A. niger* colony depending on carbon source complexity

**Figure 1:**
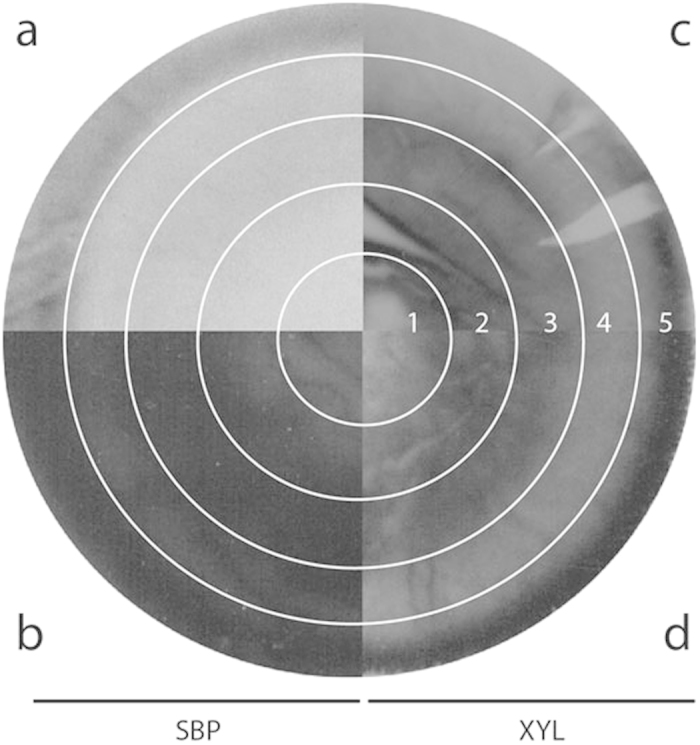
Protein synthesis and secretion. Protein synthesis (**a,c**), and protein secretion (**b**,**d**) in 5-day-old- sugar beet pulp- and xylose-grown sandwiched colonies (respectively left and right) of *A. niger*. Protein synthesis and protein secretion were localized by incorporation of 14C-labeled amino acids. Secreted proteins were detected by placing a protein binding PVDF membrane under the colony. Lane numbers indicate the concentric wells of the zone plate, lanes 1 and 5 representing the most central and peripheral zones, respectively.

Sandwiched colonies of *A. niger* were grown on minimal medium, containing 1% SBP as a carbon source (see Supplementary Fig. S1 online). *A. niger* was inoculated at a central point and the mycelium extended by radial growth so that the colony centre represents the oldest hyphae and the periphery is made up of the newly formed hyphae. The fungus cannot penetrate the membrane and therefore the gene expression is limited to the colony on top of the membrane. However, extracellular proteins can cross the membrane therefore the gene expression is limited to the colony on top of the membrane. However, extracellular proteins can pass the membrane and degrade the polymeric substrates into monomers that support fungal growth, indicating an efficient diffusion of the resulting monosaccharides to the fungal colony. After 5 days growth the diameter of each colony was approximately 5.5 cm, equaling a growth rate of around 1 cm per day which is similar to that observed by Levin *et al.* using mono- and disaccharides as carbon sources^5^. Five concentric zones were arbitrarily distinguished in the sandwiched colony. Zone 1 (Z1) was the innermost and oldest part of the colony, with a diameter of 1.5 cm. Zone 2 (Z2) consisted of the part of the colony between 1.5 and 2.5 cm, zone 3 (Z3) between 2.5 and 3.5 cm, zone 4 (Z4) between 3.5 and 4.5 cm, and zone 5 (Z5) between 4.5 and 5.5 cm. The outer border of zone 5 constituted the actively extending edge of the colony at day 5. The spatial distribution of protein synthesis and protein secretion across the colony was determined by labeling with a mixture of 14C-amino acids, either before (for synthesis) or after (for secretion) washing the fungal mycelium (Fig. 1). During growth on xylose, protein secretion occurred at the periphery of the colonies only (Fig. 1a) whereas protein synthesis occurred throughout the entire colony (Fig. 1b). Interestingly, when *A. niger* was grown on sugar beet pulp, both protein synthesis and secretion occurred throughout the colony (Fig. 1c,d). The clear difference observed between protein secretion on monosaccharides (xylose, maltose) and on the complex carbon sources comprising sugar beet pulp led us to further study the influence of the carbon source on the physiology of the mycelial colony.

### Genes are differentially expressed across the *A. niger* colony grown on sugar beet pulp

**Figure 2:**
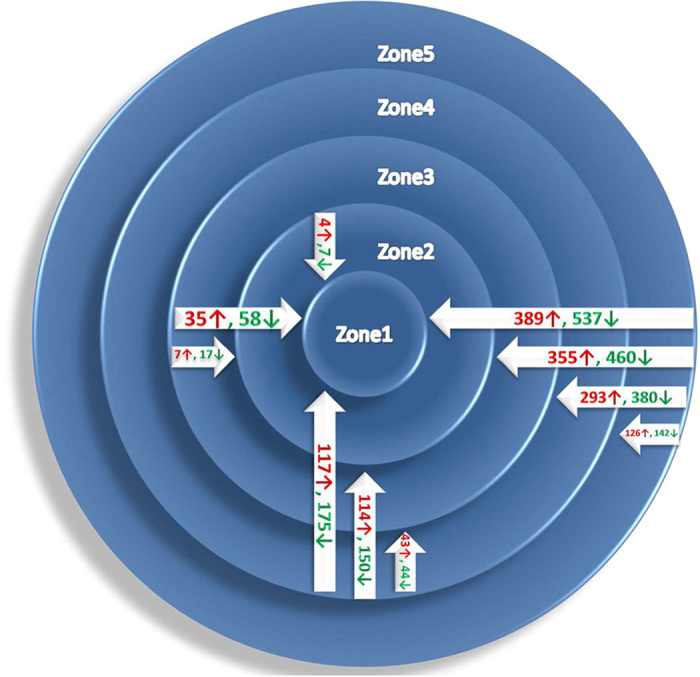
Distribution of differentially expressed genes.

To determine genome-wide gene expression differences across the *A. niger* colony grown on sugar beet pulp we used hybridization of probes derived from mRNA from the five concentric zones to Affymetrix *A. niger* GeneChips (see Materials and methods). Genome-wide principal component analysis (PCA) of gene expression on duplicate samples showed that the five concentric zones can be clearly distinguished into distinct groups corresponding to spatial locations (see Supplementary Fig. S2 online). The total gene expression sets were compared pairwise through zone 5 to zone 1 using one-way ANOVA. While ~92% (13298) of the genes showed constant expression levels throughout the colony, 1144 genes were differentially expressed (p < 0.05 and fold change >=2). Furthermore, the distribution of differentially expressed genes showed a clear spatial-related pattern: the further the physical distance between the zones, the greater the number of differentially expressed genes and the larger the average fold changes in gene expression (Fig. 2, see Supplementary Table 1, and Supplementary Fig. S3 online). The highest number and average fold change values were found in the comparison of the most peripheral zone 5 with the central zones, particularly zone 1. The most distinguishing comparison among neighboring zones was found between zone 5 and zone 4.

### Plant polysaccharides degrading genes are expressed with a different pattern across the colony

#### Pectin (43 genes)

Pectin is the most diverse and complex plant cell wall polysaccharide and requires several enzymatic activities (glycoside hydrolase, polysaccharide lyase and carbohydrate esterase) to be converted into monosaccharides. As sugar beet pulp contains up to 32% pectin, it is a good inducer of pectinase production. In the *A. niger* CBS 513.88 genome, 62 genes from 14 enzyme families are predicted as pectinolytic genes^9^. In this study, 43 pectinolytic genes (69%) showed significant expression (Table 1). Genes from most of the pectinolytic CAZy families are expressed including the five activities present in the GH28 family (PGA, PGX, RHG, RGX, XGH). The expression profiles differ from the center (Z1) to the periphery (Z5) and can be divided into 3 categories. 23 genes showed decreased expression from Z1 to Z5, 12 genes showed constant expression and 8 genes showed increased expression from Z1 to Z5. Both endo-activities and exo-activities are present in the different zones of the colony as well as genes targeting the different structures of pectins. Thereby, different isoforms are present or different genes encoding the same enzymatic activity are expressed throughout the mycelium. For example two genes encoding endoarabinanase (GH43) are highly expressed in the central part of the colony, whereas two other GH43 ABNs show a constant expression throughout the five zones. Similarly, one gene encoding a pectin lyase (PL1) is highly expressed at the centre of the colony, two PL1s are highly expressed at the periphery and one PL1 is constantly expressed throughout the colony. Thus isoforms of pectin lyases and endo-1,5-alpha-arabinanase were expressed throughout the whole colony. GH28 contains several members, for which the expression profiles can be divided into the three categories, highly expressed at the centre, highly expressed at the periphery of the colony or expressed throughout the whole colony (Table 1). Sugar beet pectin is esterified by feruloyl moieties, either as single ferulic acid substitutions or in the form of ferulic acid dehydrodimers (diFAs)^10^. In our conditions, the genes encoding feruloyl esterase A, B, and putative feruloyl esterases C, D and E showed constant expression. *faeA* and *faeE* are moderately expressed while *faeB* and *faeD* are expressed at low levels (Table 2). Remarkably, the three GH78 (α-L-rhamnosidase) showed a decrease from the centre to the periphery or constant expression throughout the colony.

#### Cellulose, xylan and xyloglucan (52 genes)

Cellulose consists of a linear polymer of glucose units connected by a β-1,4-acetal bond. The enzymes activities involved in cellulose degradation are cellobiohydrolase (CBH), β-1,4-D-glucosidase (BGL), β-1,4-D-endoglucanase (EGL) and lytic polysaccharide mono-oxygenase (LPMO). Amongst the 25 *A. niger* CBS 513.88 genes involved in cellulose degradation^11^, 21 were significantly expressed (84%). Six genes encoding BGL (GH1 and GH3) and four genes encoding EGL (GH5 and GH6) were highly expressed at the centre of the colony while one gene encoding EGL (GH12) was more highly expressed at the periphery and eight genes encoding BGL (GH3 and GH5) and two genes encoding EGL (GH12) showed constant expression throughout the mycelium. One gene encoding a LPMO (AA9, formerly GH61) was constantly expressed throughout the colony whereas two genes encoding AA9 were more highly expressed at the centre (Table 2). Thus, as described for the pectinolytic genes, different genes encoding the same enzymatic activity are present throughout the mycelium. Xylan and xyloglucan are components of hemicellulose. No less than 23 CAZy families participate in the degradation of these complex polysaccharides^12^. Thirteen genes involved in xylan and /or xyloglucan degradation are more highly expressed at the centre of the colony including two genes encoding respectively for an α-L-fucosidase (GH95) and an acetyl xylan esterases (CE1). L-fucose is a terminal sugar in xyloglucans, which are present in sugar beet pulp, although at small amounts. Despite the paucity of L-fucose in sugar beet pulp, α-L-fucosidase activity might be important to achieve complete decomposition of polysaccharides^13^. Five genes are more highly expressed at the periphery and 13 genes show a constant expression from Z1 to Z5.

#### Starch and galactomannan (9 and 7 genes)

Sugar beet is a tuber and contains two storage polysaccharides, starch and galactomannan. Starch is degraded by α-amylase (GH13), α-1,4-glucosidase (GH31) and glucoamylase (GH15). Amongst the nine genes encoding starch degrading enzymes expressed on sugar beet pulp, three were highly expressed at the periphery, three were highly expressed at the center and three were constantly expressed throughout the mycelial colony (Table 3). Galactomannan is degraded by β-1,4-mannosidase (GH2), β-1,4-endomannanase (GH5, 26), β-1,4-galactosidase (GH2, 35) and α-1,4-galactosidase (GH27, 36). Eight galactomannan related genes were expressed on sugar beet pulp, two showed elevated expression at the center, two were highly expressed at the periphery and three were constantly expressed throughout the mycelial colony (Table 4). Interestingly, α-1,4-galactosidases are also known to be active on α-galactose from xylan and therefore participate in xylan degradation.

### Genes involved in carbon metabolism are constantly expressed across the colony

Hexoses and pentoses resulting from sugar beet pulp breakdown by *A. niger* are metabolized through glycolysis, pentose catabolic pathway and pentose phosphate pathway as well as alternative glucose oxidase and dehydrogenase pathways. In our conditions, ~80% of the genes involved in these pathways showed a constant expression profile across the five zones. The same pattern was observed for the five genes of the D-galacturonic acid pathway (see Supplementary Table 2 online). Notably, genes from the L-rhamnose pathway did not show constant expression across the colony. L-rhamnose is mainly present in rhamnogalacturonan type I and type II within pectin polymers. 7.1% of the total sugar composition of sugar beet pectin is L-rhamnose^9^. Homologous gene clusters related to L-rhamnose metabolism were found by bioinformatics^14^. A L-rhamnose utilization transcription regulator in *A. niger* (RhaR, An13g00910) was later identified^15^ and a putative catabolic pathway established in *A. niger* (An03g00040, An13g00920, An13g00930 and An13g00940)^16^. Three out of the four genes involved in the putative L-rhamnose catabolic pathway showed significant differential expression with a decreasing expression profile from the centre to the periphery of the colony (Z1 to Z5). The activator RhaR remains constantly expressed throughout the colony.

To be taken up by the fungal cell, the sugars released by enzymatic hydrolysis of the sugar beet pulp polysaccharides must be transported across the plasma membrane. Interestingly, several sugar transporters were differentially expressed through the colony. Six hexose transporters were more highly expressed at the periphery whereas nine were more highly expressed at the centre of the mycelial colony (see Supplementary Table 1 online).

### Genes involved in nitrogen utilization are differentially regulated across the colony

The sugar beet pulp growth media contained 10 mM nitrate, added as the predominant nitrogen source, as well as a mixture of organic and inorganic nitrogen sources inherently present in the pulp. To determine how *A. niger* utilized the available nitrogen sources we examined expression of 199 genes with known or predicted roles in nitrogen utilization or nitrogen regulation throughout the five colony zones during growth on sugar beet pulp. 90 of the 199 genes showed at least 1.5-fold change with a confidence level of 95% between the centre of the colony (Z1) and the periphery (Z5). While the majority of nitrogen metabolic genes did not show differential expression between the centre and the periphery, 16 metabolic genes showed 1.5-fold or greater decreased expression at the periphery and 32 genes showed 1.5-fold or more increased expression at the periphery (see Supplementary Table 3 online). Approximately half of the transporter genes showed no difference in expression between Z1 and Z5. However, twelve of 43 transporter genes showed increased expression and nine genes showed decreased expression in zone 5 compared with the central zone 1. Although 16 transcription factors involved in regulation of nitrogen metabolic genes did not show differential expression, one transcription factor, the global nitrogen transcription activator AreA (An12g08960)^17^, showed higher expression at the colony centre.

Nitrate is the most abundant nitrogen source in sugar beet pulp media. In *A. niger*, nitrate is thought to be taken up by a single transporter, CrnA, and then converted to ammonium via nitrite by nitrate reductase (NiaD) and nitrite reductase (NiiA)^18^. We found that the *A. niger* nitrate transporter An08g05670 (*crnA*) and the nitrate utilization pathway genes An08g05610 (*niaD*) and An08g05640 (*niiA*) were up-regulated at the colony periphery, and An08g05670 and An08g05610 also showed higher expression in zones 3 and 4, compared to the centre of the colony. These observations are consistent with nitrate utilization occurring at the growing edge and outer zones of the colony, and may suggest a preference for nitrate by actively growing or new hyphae. No change in expression of the pathway-specific nitrate transcription activator ortholog An18g02330 (*nirA*) was observed, consistent with increased expression of nitrate utilization genes occurring in response to increased *crnA* nitrate transporter expression and increased inducer (nitrate) uptake at the colony edge.

We also investigated genes involved in the utilization of other nitrogen sources. We found higher levels of expression at the periphery for An08g03200, the homolog of the *A. nidulans* high affinity ammonium transporter MepA. Increased peripheral expression was also observed for predicted proline, urea and allantoin transporters, An11g06150 (orthologous to *A. nidulans prn*B^19^, An01g03790 (orthologous to *A. nidulans ure*A^20^ and An08g06240 (orthologous to *A. nidulans fur*A^21^, suggesting that these compounds are either being utilized in addition to nitrate or actively scavenged at the colony edge. In contrast, several nitrogen signaling genes, including the autophagy genes An04g03950 (*atg1*) and An07g10020 (*atg8*)^22^ and An11g11320 (*atg4*)^23^, showed increased expression in the centre of the colony. An02g14410, the ortholog of the major low affinity, high capacity ammonium transporter MeaA^24^,^25^ also showed elevated expression in zone 1.

### Genes related to fungal growth with a high differential expression across the colony

Interestingly, the gene An04g03980 encoding a protein very similar to a C2H2-type zinc finger protein Mhy1p showed a 2.5 fold decrease in zone 5. Mhy1p in *Yarrowia lipolytica* regulates filamentous growth and is involved in cell differentiation^26^. Moreover, one chitin synthase was more highly expressed at the periphery where the mycelium is newly formed but four chitin synthases were more highly expressed at the centre of the colony where the mycelium is older (see Supplementary Table 1 online). The thioredoxin reductase TrxB (An01g02500) was highly expressed through the five zones with an increased expression at the periphery. The expression and production of thioredoxin reductase is known to be induced on starch^27^. *A. nidulans* TrxB is involved in metabolic activity and constitutively active during germination^28^.

### Genes encoding proteases are more highly expressed at the periphery of the colony

Sixty-two (putative) extracellular proteases were predicted by orthology search with the data from Budak *et al.*^29^ (see Supplementary Table 4 online). Thirty genes were evenly expressed across the colony and eighteen genes were not significantly expressed. However, fourteen out of the sixty-two genes showed a higher expression at the periphery. The most significantly differentiated gene encodes the aspartic protease protG^5^,^30^ (An12g03300). Other genes encoding characterized proteases, are pepA^31^ (An14g04710), An02g04690^32^, protF(An03g05200), pepF^33^ (An07g08030), protA^34^ (An08g04490), protB^5^ (An08g04640) and pepB (An01g00530). These results confirm that the expression of the genes encoding for protein degradation is strongly influenced by the composition of the medium as previously described by Levin *et al.*^5^.

### The secreted protein profile correlates with measured enzyme activities

**Figure 3:**
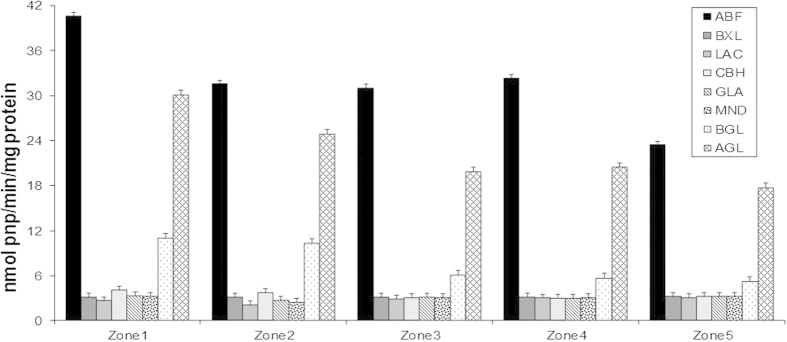
Cellulases and hemicellulases activities.

**Figure 4:**
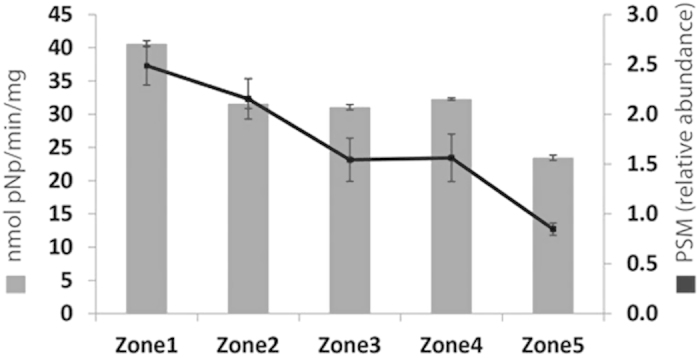
α-L-Arabinofuranosidase activity and abundance in the five zones.

Sugar beet pulp is a good inducer of protein production due to its rich content in polysaccharides. We investigated the secreted proteins from the five zones using proteomics and by measuring enzyme activities. Amongst the 146 proteins identified, 31 are pectinases, 21 are hemicellulases and 4 are cellulases (see Supplementary Table 5 online). Moreover, we measured the activities of six enzymes related to plant cell wall degradation: α-arabinofuranosidase (ABF), β-xylosidase (BXL), β-galactosidase, (LAC), β-cellobiohydrolase (CBH), glucoamylase (GLA), mannosidase (MND), β-glucosidase (BGL) and α-galactosidase (AGL) (Fig. 3). The profile of the measured enzyme activities through the five zones of the colony correlates to the abundance profile of the corresponding secreted enzymes detected in the supernatant. While most enzyme activities showed a constant profile across the colony the two highest activities measured, α-arabinofuranosidase and α-galactosidase decreased from the centre to the periphery. In *A. niger*, seven genes encode α-galactosidase An06g00170 (*aglA*), An02g11150 (*aglB*), An01g01320, An14g01800, An11g06330, An09g00260/ An09g00270 (*aglC*) and An04g02700^11^, four genes encode α-arabinofuranosidase An01g00330, An08g01710, An09g00880 (*abfA*) and An15g02300 (*abfB*)^35^,^36^ . The proteins encoded by the An15g02300 and An06g00170 genes showed decreased abundance from Z1 to Z5 and correspond with α-arabinofuranosidase activity (Fig. 4).

## Discussion

The separation of a mycelial colony into five concentric zones allows comparison of the young mycelium exploring fresh sugar beet pulp with the older mycelium growing on partially utilized sugar beet pulp. Utilization of sugar beet pulp, as a complex substrate, requires a diverse array of plant cell wall degrading enzymes. Many of the genes encoding these enzymes showed differential expression patterns across the mycelial colony. The patterns of expression for each enzyme family were likely dictated by both substrate availability and the age of the mycelium (young or older mycelium). For instance, of the four genes encoding α-L-arabinofuranosidase, one showed the highest expression at the centre of the colony while a second showed the highest expression at the periphery, and the two other genes were not significantly expressed in our experimental conditions. The result of this differential regulation is expression of Abf activity throughout the colony; however the Abf substrate specificity and enzyme activity may differ in different zones. Our analysis of α-L-arabinofuranosidase activity showed decreased activity from the inner zone to the outer zone of the colony correlating with reduced abundance of the protein detected from zone 1 to zone 5. This differential regulation of gene expression and the corresponding changes in enzyme activity highlight the ability of different parts of the *A. niger* colony to adapt to heterogeneous substrates. Surprisingly, in our experimental conditions, the feruloyl esterase B was constantly but lowly expressed throughout the colony. FaeB activity was initially identified from the supernatant of *A. niger* grown in sugar beet pulp and the production of the enzyme has been shown to be induced by pectin^37^,^38^.

Protein synthesis and protein secretion were not localized in a particular zone during growth on sugar beet pulp but occur throughout the colony. Our results contrast starkly with a similar study conducted on the simple sugars xylose or maltose^5^, where protein secretion occurred only at the periphery of the colony. Levin and coworkers’ results correlated with carbon limitation in the inner zones of the mycelial colony grown on monosaccharides and disaccharides. In contrast, carbon nutrients are not limiting on sugar beet pulp. Furthermore, microarray analysis of the transcripts expressed in the five colony zones revealed that although genes encoding plant cell wall degrading enzymes are expressed throughout the colony, specific genes show differential expression between the different growth zones.

Contrasting with the differential expression of plant cell wall degrading enzyme genes, the expression levels of genes involved in central carbon metabolism, including the hexose and pentose catabolic pathways, were similar across the colony. Other pathway specific genes, such as those of the D-galactose catabolic pathway were also expressed at similar levels throughout the colony. The constant expression of these core carbon metabolic genes is consistent with the efficient release of monosaccharides from sugar beet pulp by cell wall degrading enzymes. One exception is the L-rhamnose pathway genes, which are more highly expressed in the colony centre. The genes encoding for α-L-rhamnosidases (GH78), that release L-rhamnose from sugar beet pulp, showed the same expression profile, suggesting an adapted response to locally available monosaccharides. In addition to carbon metabolism being fine-tuned for the sugar beet media we saw differential expression of nitrogen metabolism genes. The genes required for use of the most abundant nitrogen source, nitrate, were more highly expressed at the periphery, consistent with nitrate utilization by the extending hyphae, however the expression of certain genes for uptake of alternative nitrogen sources at the periphery suggests foraging for proline, urea and allantoin from the unexplored substrate. The same trend of elevated nitrate utilization gene expression at the periphery was observed on xylose and on maltose, where no significant decrease in nitrate was detected in the colonized portion of the growth media (Levin *et al.* 2007), confirming the spatial expression of these genes is carbon source-independent. In contrast to xylose or maltose, the availability of the carbon source from sugar beet pulp is not limited during the five days of growth. The local composition of the substrate is certainly modified by the fungus. Depletion of some nutrients in the central zone where the consumption of the medium by the mycelium is the highest together with an evenly active carbon metabolism across the colony, illustrate the richness of sugar beet pulp as a nutrient source.

Our analysis of gene expression showed the differential regulation of many genes encoding enzymes across the fungal colony. While genes encoding the same or related enzymatic activity showed differential expression across the colony, the combination of these genes resulted in the enzyme activity being present throughout the whole mycelium. However, as the specific properties of the iso-enzymes (e.g. substrate specificity, pH optimum and stability) may differ, hydrolysis of different linkages of the substrate may occur in different zones. Thus, it can be concluded that *A. niger* differentially uses the enzymatic tools in its repertoire to adapt its metabolism as it colonizes complex environments. These insights participate in understanding sequential steps in saccharification of plant biomass using *A. niger* commercial enzymes and may further be used to improve enzymatic cocktails.

## Methods

### Gene annotation

Annotation of the *A. niger* open reading frames (ORFs) has been described by Pel *et al.*^39^. The Supplementary information present the ORF names of genes whose expressions have been described in our article.

### Growth conditions

*A. niger* N402 (cspA1)^40^ was grown at 30 °C under constant light in water-saturated air. Colonies were grown as a sandwiched culture^41^ in 9-cm petri dishes in a 0.2-mm thin layer of 1.25% agarose in between two perforated polycarbonate membranes (diameter, 76 mm; pore size, 0.1 μm; Osmonics, GE Water Technologies, Trevose, PA) placed on top of solidified (1.5% agar) minimal medium (7) with 1% sugar beet pulp as a carbon source or in liquid minimum medium contained in a ring plate. A ring plate consists of a polycarbonate disc (9 cm in diameter, 1.2 cm thick) with six ring-shaped wells. The inner two rings are collectively called ring 1 because of their small volume, and the outer ring is called ring 5. The wells are separated by 0.1 cm and are 0.5 cm deep and 0.5 cm wide.

Sandwiched cultures were inoculated with 1.5 μl of spore suspension (10^8^ spores/μl) and harvested after 5 days of growth (when the mycelial colony reaches the edge of the petri dish). The spore suspension concentration was estimated using a Malassez counting chamber. For each replicate, the mycelium and medium of four colonies were pooled.

### Detection of growth, protein synthesis, and protein secretion

Growth, protein synthesis, and protein secretion were monitored as described previously^42^. Sandwiched colonies were labeled with 185 kBq of [14C]N-acetylglucosamine (specific activity, 2.04 gBq/mmol; Amersham Biosciences, United Kingdom) for 10 min to detect growth. Protein synthesis and secretion were monitored by labeling with 185 kBq of a mixture of 14C-labeled amino acids (specific activity, 189 gBq/milliatom; Amersham Biosciences, United Kingdom) for 4 h. When colonies were labeled on solid medium, a protein binding polyvinylidene difluoride (PVDF) membrane was placed under the sandwiched culture to immobilize the secreted proteins. Label was adsorbed to a piece of rice paper and placed on top of the sandwiched culture. In cases of colonies placed on a ring plate, the label was applied directly in the medium or adsorbed to rice paper as described above. After being labeled, colonies were fixed with 4% formaldehyde. Fixed colonies and PVDF membranes were washed three times for 60 min with either 0.44 mM N-acetylglucosamine or 1% Casamino Acids, dried, and exposed to Kodak Biomax XAR film (Kodak Industrie, France). Labeled proteins in the culture medium of ring plates were separated on 10% sodium dodecyl sulfate (SDS)-phosphonoacetic acid gels and fixed with 45% methanol, 10% acetic acid. After the gel was enhanced with Amplify (Amersham Biosciences, United Kingdom), it was dried and exposed to Kodak Biomax XAR film (Kodak Industrie, France).

### RNA isolation

Mycelium was ground using a microdismembrator (B. Braun GmBh, Melsungen, Germany), and RNA was extracted using TRIzol reagent (Invitrogen, Carlsbad, CA) according to the instructions of the manufacturer. The RNA was purified using a Nucleospin RNA cleanup kit (Macherey-Nagel GmBh, Düren, Germany). The concentration of RNA was measured at A260. The quality of the RNA was analyzed with an Agilent 2100 bioanalyzer, using an RNA6000 LabChip kit (Agilent Technology, Palo Alto, CA).

### Microarray analysis

Biotin-labeled antisense cDNA was generated by labeling 20 or 2 μg of total RNA with a BioArray high-yield RNA transcription labeling kit (ENZO) or an Affymetrix eukaryotic one-cycle target labeling and control reagent package, respectively. The quality of the cDNA was checked using the Agilent 2100 bioanalyzer. The labeled cDNA was hybridized to Affymetrix *A. niger* GeneChips (Affymetrix, Santa Clara, CA). The coding sequence of the annotated genome of CBS513.88^39^ was taken as the sequence template. Oligonucleotide probes were designed with 600-bp fragments, starting from the 3’ end of the gene. The probe sets consist of 12 pairs (match and mismatch) of 25-bp oligonucleotide probes, which are scattered across the chip. Arrays were hybridized with three independently obtained RNA samples of the peripheries of 5-day-old sandwiched cultures grown on sugar beet pulp. Microarray data has been deposited in the Gene Expression Omnibus database^43^ (Accession No. GSE66641).

Micro-array data was analyzed using the Bioconductor tool package version 2.8 (http://www.bioconductor.org/) together with house-made Perl (version.5.0) and Python (version 3.0) scripts. Utilizing the R statistical language and environment, the probe intensities were normalized for background by the robust multiarray average (RMA) method which makes use of only the perfect match (PM) probes^44^. Further, normalization was processed by quantiles algorithm, and with the medianpolish summary method the gene expression values were calculated from the PM probes. Genes with expression value lower than 15 (median) were considered not significantly expressed and the ones higher than 170 (top 10%) were considered highly expressed (see Supplementary Table 6, online).

Further statistical analyses were applied to the normalized gene expression data using the CyberT tool package. BayesAnova tests were performed on each gene through paizone carbon sources. Adjusted cut-off value of *P* < 0.05 and fold change >=2 was used to determine the statistical significance of gene expression difference.

### Gene orthology search, extracellular protease prediction, expression clustering and visualization

Gene orthology search was performed by OrthoMCL^45^ by the parameters of E-value 1E^−10^, inflation level 1 and sequence coverage 40%. Extracellular proteins were predicted by combining Phobius^46^, SignalP^47^, PrediSi^48^, CELLO^49^, MultiLOC^50^ and WoLF-PSORT^51^ with majority votes. Default settings of each SCL predictor were used, with the species parameter as “Eukaryotic” or “Fungi”. Putative proteases, inclusive the inhibitor information, were retrieved by AspGD gene annotation repository and literature researches. Protein functional domain prediction was performed by HMMER v.3.0^52^ using the complete Pfam-A and Pfam-B models (data retrieved from Pfam database, version November 2012) with the trust cutoff and the gathering cutoff. The resulting Pfam predictions were pooled. Hierarchical clusters were made using the normalized expression data from selected genes by calculating the pearson’s correlation distances. Clusters and expression correlation profiles were visualized by Genesis^53^. Genes with expression value lower than 50 were colored dark blue, the ones higher than 1000 were colored red and the values in-between were colored gradient between these 2 colors.

### Proteomics

#### Protein separation and digestion

Samples were 4 times concentrated using a vacuum concentrator (Thermo Scientific, Bremen) and 30 μl of each of the samples were run on a 12% Bis-Tris 1D SDS-PAGE gel (Biorad) for 2–3 cm and stained with colloidal coomassie dye G-250 (Gel Code Blue Stain Reagent, Thermo Scientific). The lane was cut into two bands, which were treated with 6.5 mM dithiothreitol (DTT) for 1 h at 60 °C for reduction and 54 mM iodoacetamide for 30 min for alkylation. The proteins were digested overnight with trypsin (Promega) at 37 °C. The peptides were extracted with 100% acetonitrile (ACN) and dried in a vacuum concentrator.

#### Mass spectrometry: RP-nanoLC-MS/MS

The data was acquired using an LTQ-Orbitrap Discovery coupled to an Agilent 1200.

Peptides were first trapped (Dr Maisch Reprosil C18, 3 um, 2 cm × 100 um) before being separated on an analytical column (50 um × 400 mm, 3 um, 120 Å Reprosil C18-AQ). Trapping was performed at 5 μl/min for 10 min in solvent A (0.1 M acetic acid in water), and the gradient was as follows; 10 - 37% solvent B in 30 min, 37–100% B in 2 min, 100% B for 3 min, and finally solvent A for 15 min. Flow was passively split to 100 nl/min. Data was acquired in a data-dependent manner, to automatically switch between MS and MS/MS. Full scan MS spectra from m/z 350 to 1500 were acquired in the Orbitrap at a target value of 5^e5^ with a resolution of 30,000 at m/z 400. The five most intense ions were selected for fragmentation in the linear ion trap at a normalized collision energy of 35% after the accumulation of a target value of 10,000. The data was normalized for volume per mg biomass.

#### Data analysis

Raw files were processed using Proteome Discoverer 1.3 (version 1.3.0.339, Thermo Scientific, Bremen, Germany). Database search was performed using *A. niger* Mascot (version 2.4.1, Matrix Science, UK) as the search engine. Carbamidomethylation of cysteines was set as a fixed modification and oxidation of methionine was set as a variable modification. Trypsin was specified as enzyme and up to two miss cleavages were allowed. Data filtering was performed using percolator, resulting in 1% false discovery rate (FDR). Additional filters were; search engine rank 1 peptides and Mascot ion score >20. Raw files corresponding to one sample were merged into one result file. The mass spectrometry proteomics data have been deposited to the ProteomeXchange Consortium^54^ via the PRIDE partner repository with the dataset identifier PXD002130.

## Additional Information

**How to cite this article**: Benoit, I. *et al.* Spatial differentiation of gene expression in *Aspergillus niger* colony grown for sugar beet pulp utilization. *Sci. Rep.* **5**, 13592; doi: 10.1038/srep13592 (2015).

## Supplementary Material

Supplementary Information

Supplementary Table 1

Supplementary Table 2

Supplementary Table 4

Supplementary Table 5

Supplementary Table 6

## Figures and Tables

**Table 1:** Pectinolytic genes.

UniqueID	Annotation	CAZy family	Fold change R5 over R1
Higher expression at the centre of the colony			
An03g02080	putative exopolygalacturonase pgaX	GH28	0.063
An03g06740	putative exo-alpha 1,4-polygalacturonase PGX1	GH28	0.092
An12g00950	rhamnogalacturonase rhgA	GH28	0.121
An05g02440	polygalacturonase III precursor pgaC	GH28	0.126
An11g04040	putative exo-alpha 1,4-polygalacturonase PGX1	GH28	0.330
An01g14670	polygalacturonase E precursor pgaE	GH28	0.422
An18g04810	putative exo-polygalacturonase PGX from patent WO9414966-A	GH28	0.475
An04g09700	putative endo-xylogalacturonan hydrolase xghA	GH28	0.482
An01g11520	polygalacturonase pgaI	GH28	0.518
An01g14650	putative exo-alpha 1,4-polygalacturonase PGX1	GH28	0.549
An14g05820	putative beta-galactosidase lacA	GH35	0.303
An06g00290	putative beta-galactosidase lacA	GH35	0.337
An09g01190	endo 1,5-alpha-arabinanase abnA	GH43	0.388
An02g10550	putative endo-alpha-1,5-arabinanase abnA	GH43	0.264
An08g01710	putative alpha-L-arabinofuranosidase abfA	GH51	0.399
An18g05940	putative arabinogalactan endo-1,4-beta-galactosidase gal1	GH53	0.385
An01g06620	putative alpha-L-rhamnosidase A precursor rhaA	GH78	0.529
An09g02160	rhamnogalacturonan acetyl esterase rgaeA	CE12	0.216
An04g09360	putative hypothetical protein CC0812	CE12	0.120
An04g09690	putative pectin methylesterase pme1	CE8	0.307
An14g01130	putative rhamnogalacturonase B precursor rhgB	PL4	0.319
An11g00390	putative rhamnogalacturonase rhiE	PL4	0.511
An15g07160	putative pectin lyase pelA	PL1	0.555
Higher expression at the periphery of the colony			
An09g03260	endo-polygalacturonase D pgaD	GH28	1.816
An02g04900	endopolygalacturonases pgaB	GH28	3.262
An15g05370	polygalacturonase pgaII	GH28	2.496
An16g06990	endo-polygalacturonase A pgaA	GH28	10.57
An01g10350	putative secreted beta-galactosidase lacA	GH35	2.409
An01g00330	alpha-l-arabinofuranosidase a precursor abfA	GH51	2.951
An03g06310	pectin methylesterase pmeA	CE8	4.101
An03g00190	pectin lyase pelB	PL1	2.046
An19g00270	pectin lyase pelD	PL1	4.322
Constant expression through the colony			
An12g07500	putative exopolygalacturonase pgaX	GH28	0.672
An02g12450	putative polygalacturonase XOPG1	GH28	0.967
An06g02070	putative rhamnogalacturonase rhgA	GH28	1.094
An14g04200	rhamnogalacturonase rhgB	GH28	1.146
An01g12150	beta-galactosidase lacA	GH35	0.749
An16g02730	putative endo 1,5-alpha-arabinase abnA from patent EP506190-A	GH43	0.725
An02g01400	putative endo-alpha-1,5-arabinanase abnA	GH43	0.863
An15g02300	arabinofuranosidase B abfB	GH54, CBM42	0.823
An04g09070	putative alpha-L-rhamnosidase ramA	GH78	0.783
An18g04800	putative alpha-L-rhamnosidase A precursor rhaA	GH78	1.223
An14g02920	putative hypothetical conserved protein yesR	GH105	0.708
An14g04370	pectin lyase pelA	PL1	1.094

**Table 2:** Cellulose, xylan and xyloglucan degrading genes.

UniqueID	Annotation	CAZy family	Fold change R5 over R1
Higher expression at the centre of the colony			
An11g02100	putative furostanol glycoside 26-O-beta-glucosidase CSF26G1	GH1	0.229
An03g03740	putative beta-glucosidase bgl4	GH1	0.431
An17g00520	putative beta-glucosidase precursor BGLUC	GH3	0.158
An11g06080	putative beta-glucosidase 1 bgl1	GH3	0.301
An14g01770	putative beta-glucosidase bgln	GH3	0.511
An17g00300	putative bifunctional xylosidase-arabinosidase xarB	GH3	0.625
An01g11670	putative endo-beta-1,4-glucanase A eglA	GH5, CBM1	0.061
An07g08950	endoglucanase B eglB	GH5	0.158
An08g01760	putative cellulase from patent WO9733982-A1	GH6	0.287
An15g04550	putative xylanase A xynA from patent WO200068396-A2	GH11	0.519
An01g00780	endo-1,4-xylanase xynB	GH11	0.564
An01g03340	putative xyloglucan-specific endo-beta-1,4-glucanase	GH12	0.570
An06g00170	alpha-galactosidase aglA	GH27, CBM13	0.620
An14g05820	putative beta-galactosidase lacA	GH35	0.303
An06g00290	putative beta-galactosidase lacA	GH35	0.337
An02g00140	putative xylan 1,4-beta-xylosidase xynB	GH43	0.620
An08g01710	putative alpha-L-arabinofuranosidase abfA	GH51	0.399
An08g05230	putative hypothetical endoglucanase IV	GH61	0.506
An01g01870	putative hypothetical Avicelase III aviIII	GH74, CBM1	0.239
An16g00540	putative large secreted protein	GH95	0.566
An14g02670	putative endoglucanase IV egl4	AA9	0.314
An15g04570	putative endoglucanase IV	AA9	0.446
An12g05010	acetyl xylan esterase axeA	CE1	0.253
An07g03100	putative Esterase E	CE1	0.5825
Higher expression at the periphery of the colony			
An03g05380	similarity to cellulase FI-CMCase	GH12	1.720
An01g01320	putative alpha-galactosidase agal	GH27	1.586
An01g10350	putative secreted beta-galactosidase lacA	GH35	2.409
An09g00260	alpha-galactosidase aglC	GH36	7.282
An09g00270	alpha-galactosidase aglC	GH36	9.079
An01g00330	alpha-l-arabinofuranosidase a precursor abfA	GH51	2.952
Constant expression through the colony			
An18g03570	beta-glucosidase bgl1	GH3	0.863
An15g01890	putative beta-glucosidase precursor bgl2	GH3	0.918
An01g09960	xylosidase xlnD	GH3	1.069
An08g01100	putative exo-1,3-beta-glucanase KlEXG1	GH5	0.732
An16g06800	putative endoglucanase eglB	GH5, CBM1	0.759
An16g02100	putative hypothetical protein YIR007w	GH5	0.796
An03g01050	putative endo-beta-1,4-glucanase	GH5	0.975
An11g07660	putative exo-1,3-beta-glucanase Xog	GH5	1.193
An03g00940	endo-1,4-beta-xylanase A precursor xynA	GH10	1.015
An01g14600	putative endo-1,4-beta-xylanase B xynB from patent WO9414965	GH11	0.848
An14g02760	endoglucanase A eglA	GH12	1.170
An03g05530	putative endo-beta-1,4-glucanase EGIII-like from patent WO9931255-A2	GH12	1.209
An02g11150	alpha-galactosidase aglB	GH27	0.767
An14g01800	putative alpha-galactosidase	GH27	0.711
An09g03300	putative alpha-xylosidase XylS	GH31	1.218
An01g12150	beta-galactosidase lacA	GH35	0.749
An11g03120	similarity to endo-1,4-beta-xylanase XynD	GH43	0.720
An08g10780	putative hypothetical protein T16K5.230	GH43, CBM35	0.697
An15g02300	arabinofuranosidase B abfB	GH54, CBM42	0.823
An03g00960	1,4-beta-D-arabinoxylan arabinofuranohydrolase axhA	GH62	0.919
An14g05800	alpha-glucuronidase aguA	GH67	0.780
An15g04900	putative endoglucanase IV egl4	AA9	0.910
An09g00120	ferulic acid esterase A faeA	CE1	0.941

**Table 3:** Starch degrading genes.

UniqueID	Annotation	CAZy Family	Fold change R5 over R1
Higher expression at the centre of the colony			
An01g06120	putative 4-alpha-glucanotransferase/amylo-1,6-glucosidase Gdb1	GH13	0.434
An02g13240	putative alpha-1-6-glucosidase glcA	GH13	0.546
An01g04880	putative alpha-glucosidase II	GH31	0.210
Higher expression at the periphery of the colony			
An11g03340	acid alpha-amylase	GH13	4.037
An01g10930	putative enzyme with sugar transferase activity from patent JP11009276-A	GH31	1.740
An04g06920	extracellular alpha-glucosidase aglU	GH31	5.480
Constant expression through the colony			
An04g06930	putative extracellular alpha-amylase amyA/amyB	GH13	0.903
An03g06550	glucan 1,4-alpha-glucosidase glaA	GH15, CBM20	1.299
An09g05880	putative alpha-glucosidase ModA	GH31	1.481

**Table 4:** Galactomannan degrading genes.

UniqueID	Annotation	CAZy Family	Fold change R5 over R1
Higher expression at the centre of the colony			
An06g00170	alpha-galactosidase aglA	GH27, CBM13	0.612
An05g01320	putative mannase man1	GH5	0.651
Higher expression at the periphery of the colony			
An01g01320 An09g00260	putative alpha-galactosidase agal	GH27	1.586
An09g00270	alpha-galactosidase aglC	GH36	9.079
Constant expression through the colony			
An14g01800	putative alpha-galactosidase	GH27	0.710
An02g11150	alpha-galactosidase aglB	GH27	0.766
An04g02700	alpha-galactosidase	GH36	0.942
