# Supplementary material for: Spatial differentiation of gene expression in Aspergillus niger colony grown for sugar beet pulp utilization
Source: Sci Rep. 2015 Aug 28;5:13592. doi: 10.1038/srep13592 (PMC4552001; doi:10.1038/srep13592)
Supplement: Supplementary Information [file srep13592-s1.pdf]

Supplementary information for

Spatial differentiation of gene expression in *Aspergillus niger* colony grown for sugar beet pulp utilization

Isabelle Benoit[1,2,3*,+], Miaomiao Zhou[1,3,+], Alexandra Vivas Duarte[1,2,3], Damien J. Downes[4], Richard B. Todd[4], Wendy Kloezen[2], Harm Post[5], Albert J. R. Heck[5], A. F. Maarten Altelaar[5], Ronald P. de Vries[1,3]

[1]Fungal Physiology, CBS-KNAW Fungal Biodiversity Centre, Uppsalalaan 8, 3584 CT Utrecht, The Netherlands.
[2]Microbiology & Kluyver Centre for Genomics of Industrial Fermentations, Utrecht University, Padualaan 8, 3584 CH, Utrecht, The Netherlands.
[3]Fungal Molecular Physiology, Utrecht University, Utrecht, The Netherlands.
[4]Department of Plant Pathology, Kansas State University, 4024 Throckmorton Plant Sciences Center, Manhattan, KS 66506, USA.
[5]Biomolecular Mass Spectrometry and Proteomics, Bijvoet Centre for Biomolecular Research and Utrecht Institute for Pharmaceutical Sciences, Utrecht University, Padualaan 8, 3584 CH Utrecht, The Netherlands.

For correspondence: i.benoit@cbs.knaw.nl

Figure S1: Ring plate. Ring plate system containing minimum medium for Aspergillus and 1% sugar beet pulp. A porous polycarbonate membrane (diameter 76 mm, thickness 5–10 µm, 6×108 pores cm−2, pore size 0.1 µm; GEOsmonics, USA) was placed on top of the ring plate and 1.5 µl of spore suspension (108 spores µl−1) of *A. niger* strain N402 (Bos et al., 1988) was inoculated in the center. After 12h, a second polycarbonate membrane was placed on top of the first membrane to prevent sporulation (A). After 5 days of growth, the mycelium and the culture medium of each ring were harvested (B, C).

Figure S1: Genome scale PCA analysis with gene expression of the five zones.

Figure S2: Distribution of differentially expressed genes.

Table S1: Differentially expressed genes through the five zones.

Table S2: Expression of genes involved in central sugar metabolism of *A. niger*. The significantly differentially expression are marked out, the ones that are higher expressed in periphery are marked red with an asterisk (*), and the ones are higher in centre are marked green with asterisk (*). Cutoff values: fold change >2, P-value< 0.05.

Table S3. Nitrogen metabolism, nitrogen transport and autophagy genes.

Table S4: Secreted protein profile through the five zones.

Figure S1: Ring plate.

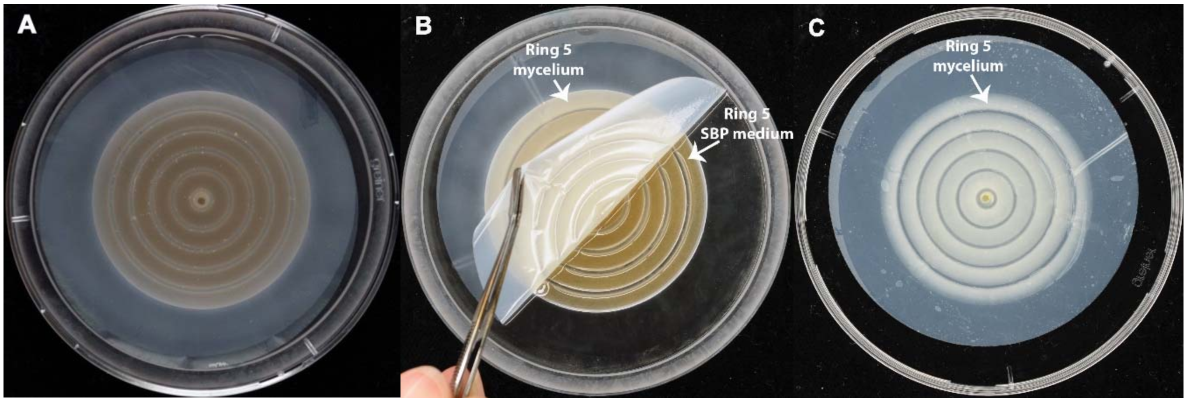

Figure S2: Genome scale PCA analysis with gene expression of the 5 zones.

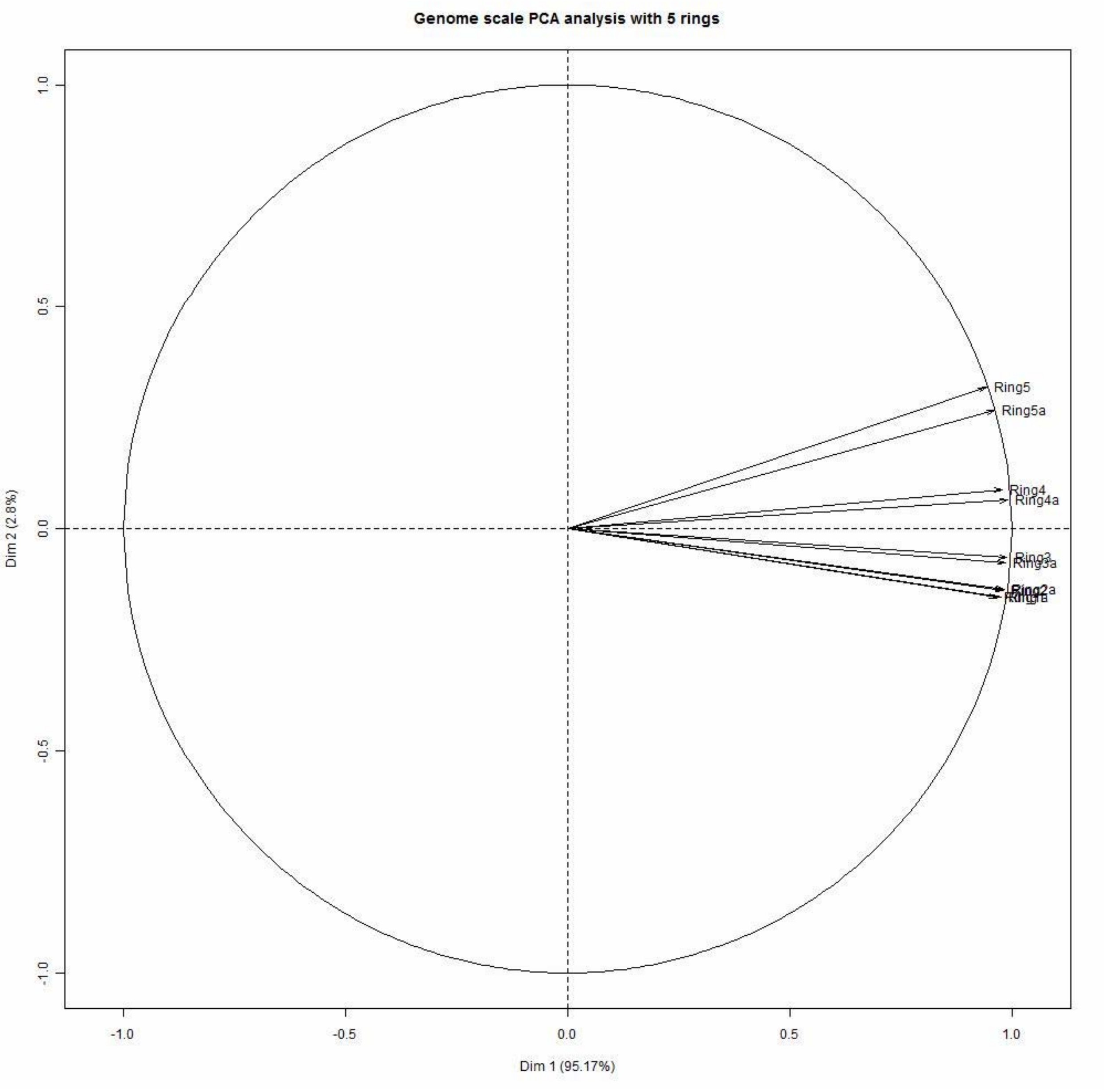

Figure S3: Distribution of differentially expressed genes.

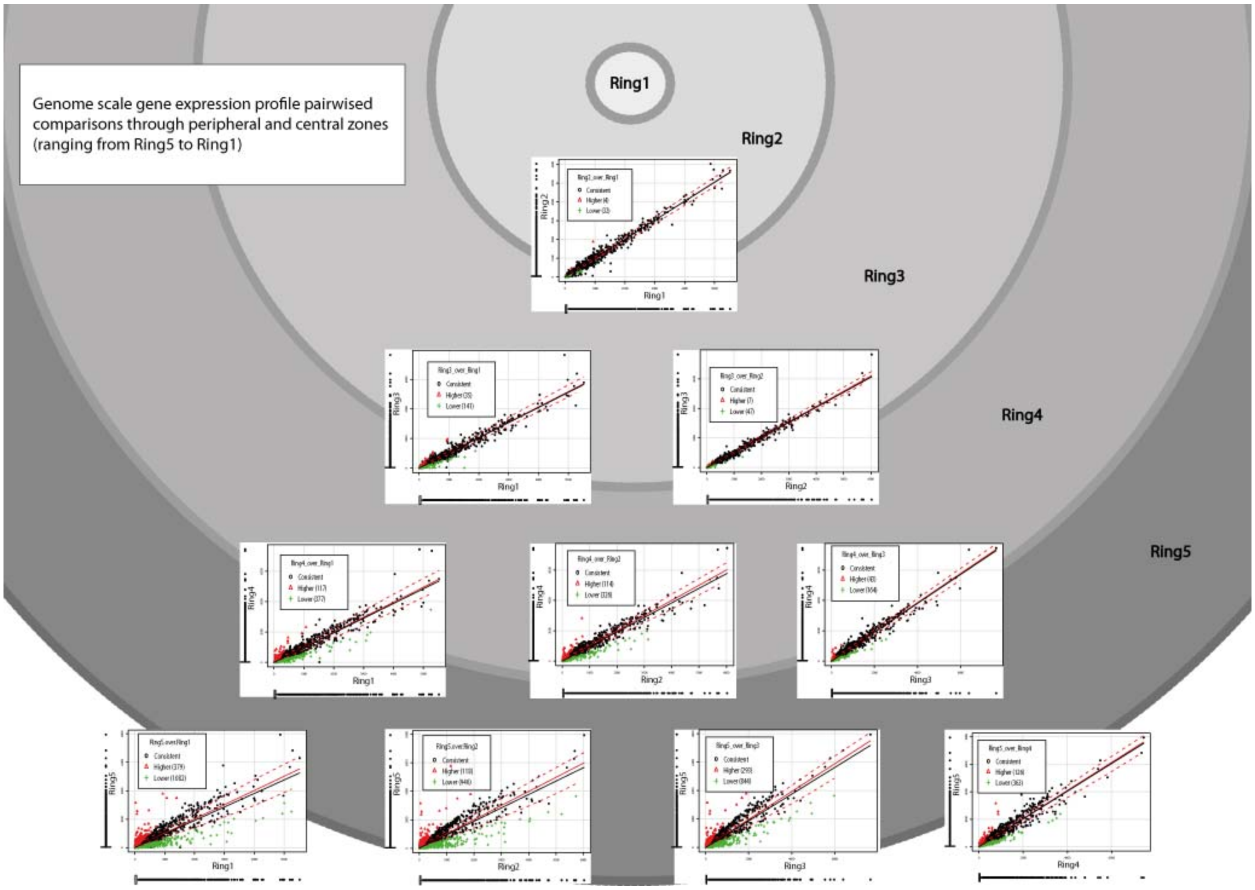

Table S3. Nitrogen metabolism, nitrogen transport and autophagy genes.

UniqueID	Annotation	Fold change R5 over R1
	**Higher expression at the centre of the colony**	
An17g00910	Gamma-aminobutyrate transaminase	0.363
An15g02590	Has domain(s) with predicted aminomethyltransferase activity, oxidoreductase activity, role in glycine catabolic process, oxidation-reduction process and cytoplasm localization	0.416
An02g08270	Has domain(s) with predicted oxidoreductase activity and role in oxidation-reduction process	0.432
An01g13810	Urea carboxylase	0.444
An11g02200	Putative 4-hydroxyphenylpyruvate dioxygenase	0.457
An14g02460	Flavohemoglobin *fhbA*	0.484
An02g06030	Urate oxidase *uaZ*	0.487
An01g12090	Putative benzonitrilase	0.518
An04g03890	Ortholog(s) have role in purine nucleobase catabolic process	0.567
An07g09920	Glutamate synthase (NADH)	0.576
An03g06870	Cytidine deaminase	0.580
An11g02180	Ortholog(s) have homogentisate 1,2-dioxygenase activity and role in L-phenylalanine catabolic process, tyrosine catabolic process	0.596
An01g12570	Putative histidine biosynthesis trifunctional protein; induced by growth on starch and lactate	0.636
An01g01530	Proline dehydrogenase	0.651
An04g07200	Putative kynureninase	0.645
An01g14100	Putative ERAD component *mifA*	0.635
An13g01860	Has domain(s) with predicted amino acid transmembrane transporter activity, role in amino acid transmembrane transport and membrane localization	0.276
An13g00840	Ortholog(s) have L-lysine transmembrane transporter activity, L-proline transmembrane transporter activity, arginine transmembrane transporter activity, polyamine transmembrane transporter activity	0.456
An15g01900	Has domain(s) with predicted amino acid transmembrane transporter activity, role in amino acid transmembrane transport and membrane localization	0.448
An16g07900	Has domain(s) with predicted amino acid transmembrane transporter activity, role in amino acid transmembrane transport and membrane localization	0.494
An12g10000	Putative GABA permease *gabA*	0.530
An03g00430	Has domain(s) with predicted amino acid transmembrane transporter activity, role in amino acid transmembrane transport and membrane localization	0.631
An02g14410	Putative ammonium transport protein	0.577
An14g01850	Ortholog(s) have gamma-aminobutyric acid:proton symporter activity, putrescine transmembrane transporter activity and role in gamma-aminobutyric acid transport, putrescine transport, transmembrane transport	0.493
An15g00330	Has domain(s) with predicted amino acid transmembrane transporter activity, role in amino acid transmembrane transport and membrane localization	0.653

An07g08980	Ortholog(s) have 1-phosphatidylinositol-3-phosphate 5-kinase activity, phosphatidylinositol-3-phosphate binding activity	0.449
An04g09220	Ortholog(s) have protein kinase regulator activity	0.503
An04g03950	Autophagy-related serine/threonine kinase; essential for autophagy *atg1*	0.541
An07g10020	Putative autophagy-related ubiquitin modifier; autophagosomal membrane protein; essential for autophagy *atg8*	0.642
An11g11320	Cysteine protease; predicted role in autophagic vacuole assembly *atg4*	0.536
An03g02660	Ortholog(s) have ubiquitin-protein transferase activity and role in protein ubiquitination, ubiquitin-dependent protein catabolic process via the multivesicular body sorting pathway	0.566
An08g01060	Ortholog(s) have phosphatidylinositol binding, ubiquitin binding, ubiquitin-protein transferase activity	0.611
	**Higher expression at the periphery of the colony**	
An11g06590	Ortholog(s) have 2-hexaprenyl-6-methoxy-1,4-benzoquinone methyltransferase activity	1.555
An04g05860	Ribose-phosphate pyrophosphokinase	1.576
An12g00410	Ortholog(s) have mRNA binding activity, role in arginine catabolic process, positive regulation of cellular response to oxidative stress, positive regulation of nuclear-transcribed mRNA poly(A) tail shortening and cytosol localization	1.586
An01g07510	Putative benzonitrilase	1.602
An04g04130	Ornithine transaminase	1.642
An04g05260	Saccharopine dehydrogenase (NADP+, L-glutamate forming)	1.650
An08g03070	Aminomethyltransferase	1.679
An01g06560	Argininosuccinate lyase	1.717
An02g14520	GMP synthase	1.718
An08g04390	Ortholog(s) have glycine dehydrogenase (decarboxylating) activity, role in glycine catabolic process, one-carbon metabolic process, protein lipoylation and mitochondrion localization	1.718
An04g00430	Branched chain amino acid aminotransferase	1.786
An02g08430	Ortholog(s) have histone-arginine N-methyltransferase activity, ribosome binding activity and role in peptidyl-arginine methylation, to asymmetrical-dimethyl arginine, ribosome biogenesis	1.792
An11g06820	3'-5' bisphosphate nucleotidase	1.806
An08g05640	Putative nitrite reductase *niiA*	1.812
An02g01610	Ortholog(s) have urease activity, role in urea catabolic process and cytosol, nucleus localization	1.812
An17g01640	1-(5-phosphoribosyl)-5-[(5-phosphoribosylamino)methylideneamino]imidazole-4-carboxamide isomerase	1.856
An13g01080	ATP phosphoribosyltransferase	1.903
An11g04730	Prolyl amino peptidase involved in protein degradation; removes N-terminal proline and hydroxyproline residues from peptides *papA*	1.924
An02g13740	Putative Gly-X carboxypeptidase precursor	1.980
An15g02490	Ortholog(s) have NAD binding, homoisocitrate dehydrogenase activity, role in lysine biosynthetic	2.023

	process and mitochondrion localization	
An02g10750	Cysteine synthase	2.036
An01g11930	Histidinol-phosphate aminotransferase	2.235
An02g14660	Ortholog(s) have 90S preribosome, nucleolus, spindle localization	2.278
An04g02800	Pyrroline-5-carboxylate reductase	2.360
An02g07500	Saccharopine dehydrogenase *lys1*	2.629
An11g09780	PAPS reductase	2.752
An08g02990	Adenylylsulfate kinase	4.440
An04g01340	Putative asparagine synthase	5.157
An08g05610	Nitrate reductase *niaD*	6.398
An02g06150	Branched chain amino acid aminotransferase	7.111
An01g14960	Asparaginase	2.486
An16g02520	Threonine synthase	1.558
An08g06240	Protein with similarity to uracil transport protein FUR4 of *Schizosaccharomyces pombe*	1.710
An14g02590	Has domain(s) with predicted amino acid transmembrane transporter activity, role in amino acid transmembrane transport and membrane localization	5.159
An04g00530	Ortholog(s) have role in positive regulation of (R)-carnitine transmembrane transport, positive regulation of polyamine transmembrane transport	3.142
An16g01920	Has domain(s) with predicted amino acid transmembrane transporter activity, role in amino acid transmembrane transport and membrane localization	2.160
An16g06090	Has domain(s) with predicted amino acid transmembrane transporter activity, role in amino acid transmembrane transport and membrane localization	2.171
An02g09790	Ortholog(s) have thiamine transmembrane transporter activity, role in thiamine transmembrane transport and endoplasmic reticulum, plasma membrane localization	1.788
An08g03200	Ortholog(s) have high-affinity secondary active ammonium transmembrane transporter activity, methylammonium transmembrane transporter activity	1.811
An07g03070	Ortholog(s) have L-aspartate transmembrane transporter activity, L-glutamate transmembrane transporter activity, antiporter activity, uniporter activity	3.012
An11g06150	Ortholog(s) have L-proline transmembrane transporter activity, role in proline transport and Golgi apparatus, endoplasmic reticulum, fungal-type vacuole, plasma membrane localization	4.503
An08g05670	Putative nitrate transporter *crnA*	9.182
An15g04600	Putative sulfate permease	2.226
An01g03790	Ortholog(s) have putrescine transmembrane transporter activity, spermidine transmembrane transporter activity, urea transmembrane transporter activity and role in putrescine transport, spermidine transport, urea transport	1.648
